# Antenatal Bartter's syndrome with sensorineural deafness

**DOI:** 10.4103/0971-4065.50677

**Published:** 2009-01

**Authors:** R. P. Bhamkar, A. Gajendragadkar

**Affiliations:** Department of Pediatrics, Gurunanak Hospital, Bandra (E), Mumbai, India

**Keywords:** Antenatal, Bartter's syndrome, sensorineural deafness

## Abstract

Bartter's syndrome is a group of inherited, salt-losing tubulopathies presenting as metabolic alkalosis with normotensive hyperreninemia and hyperaldosteronism. We report here the first case of a neonate with bilateral, sensorineural deafness, a variant of antenatal Bartter's syndrome from an Indian community.

## Introduction

Bartter's Syndrome (BS) is characterized by hypokalemic, hypochloremic metabolic alkalosis with normal or low blood pressure, despite high plasma renin activity and serum aldosterone. The inheritance pattern is autosomal recessive. Antenatal BS with bilateral sensorineural deafness (BSND) was first described in children born to a consanguineous couple from a Bedouin family of Southern Israel.[1] We report here the first case of a neonate with a BSND variant of BS from an Indian community.

## Case Report

A female baby was born at 30 weeks of gestation to a healthy, consanguineous, Indian, Hindu couple by normal vaginal delivery. Antenatally, the mother had severe, unexplained polyhydramnios. The mother had had one previous, spontaneous abortion at the 10^th^ week of gestation.

The baby's birth weight was 1.31 kg. There was no craniofacial dysmorphism but the baby developed polyuria postnatally. On the seventh day of life, the baby became dehydrated and lethargic. She lost 14% of her birth weight and her urine output was up to 10 mL/kg/h.

On investigation, the hemogram, C-reactive protein, blood sugar levels, and CSF study were found to be normal. Serum electrolytes showed hyponatremia (sodium, 101 mmol/L), hypokalemia (potassium, 2 mmol/L), and hypochloremia (chloride, 52 mmol/L). Serum calcium was 6.9 mg/dL (normal, 8–11 mg/dL), serum magnesium was normal. The arterial blood gas report showed metabolic alkalosis (pH: 7.447, pCO_2_: 39.9, pO_2_: 138, HCO_3_: 27.6). Urine osmolality was 102 mOsm/kg H_2_O (Normal: 50–1400 mOsm/kg H_2_O). In the first week of life, urinary electrolytes showed renal salt-wasting in the form of hyperchloruria, hypernatriuria (Na: 76 meq/L, Chloride: 85 meq/L) but the potassium levels was normal. Blood urea nitrogen was 49 mg/dL and creatinine was 1.2 mg/dL. The child's blood pressure was normal and the renal sonogram was normal.

A diagnosis of antenatal Bartter's syndrome was considered. Plasma renin was 35 U/L (Normal: 2–10 U/L) and aldosterone was 5583.9 U/L (Normal: 150–1050 U/L), which confirmed our diagnosis. Prostaglandin levels in blood and urine were not measured in this case. Behavioral Observational Audiometry suggested a moderate to severe hearing loss. Follow-up auditory brainstem response test showed hearing loss at higher frequencies, which suggested a BSND variant of antenatal BS.

Fluids were increased to 250–300 mL/kg/day, sodium supplements were increased to 20 meq/kg/day, and potassium to 24 meq/kg/day. Subsequently, her hydration improved and the electrolyte levels became normal (Sodium, 150 meq/L and potassium, 3.6 meq/L). Eventually, the baby developed intractable sepsis and succumbed on the 22^nd^ day of life before Indomethacin therapy could be initiated. The baby's DNA analysis was planned but could not be done because of the nonavailability of the required genetic probe in India. Also, we could not retrospectively trace any positive family history for the disorder and on follow-up, we found that the couple could not conceive again even after four years of this incidence and do not have any live issue to date.

## Discussion

Bartter's Syndrome is a rare tubular channelopathy with an incidence of 1.2 per million individuals. Very few cases of Bartter's Syndrome have been reported from India and no case of the BSND variant of BS has yet been reported.[2]

In 1962, Bartter and colleagues first described hypokalemic, hypochloremic metabolic alkalosis in two children and one man.[3] Since then, many advances have occurred in better understanding the pathophysiology and genetics of the disease. Depending on the severity and age of presentation, Bartter's syndrome has been classified as: Antenatal or neonatal BS and Classic BS. Antenatal BS is characterized by *in utero* or neonatal age of presentation, presence of nephrocalcinosis, higher urinary loss of sodium, potassium, and chloride. Classical BS presents at a later age and is milder in course.

**Figure 1:**
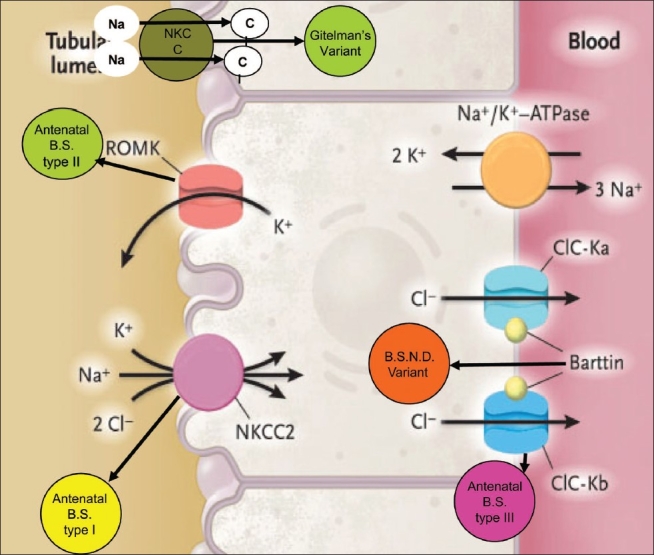
Types of channelopathies involving different channels

Based on pathophysiology and genetics, BS can be classified as [Table 1, Figure 1]:

**Table 1:** Bartter's syndrome: Different types and variants

	BS type I	BS type II	BS type III	BS type IV BSND variant	Gitelman's variant
Channel	NKCC2	ROMK	CIC-Kb	CIC-Kb/CIC-Ka	NCCT
Location	TAL	TAL, CD	TAL, DCT	TAL, Inner ear	DCT
Gene	SLC12A1	KCNJ1	CLCNKB	BSND	SLC12A3
Chromosome	15q15-21	11q24	1p36	1p31	16q13
Polyhydramnios	Present	Present	Absent	Present	Absent
Gestational age	Preterm	Preterm	Near term	Preterm	Term
Age of onset	Antenatal	Antenatal	<1year	Antenatal	6–13 years
Symptoms	Polyuria	Polyuria	Hypokalemia failure to thrive	Polyuria, deafness	Hypokalemia, tetany
Urine Ca excretion	High	High	Moderate	High	Hypocalciuria
Nephrocalcinosis	Present	Present	Usually absent	Present	Absent
Magnesium	Normal	Normal	Low or normal	Normal	Always low
Prostaglandin level	Increased	Increased	Increased	Increased	Near normal
Prostaglandin excretion	Increased	Increased	Increased	Increased	Normal

1. BS, antenatal, type I, involving defect in NaK 2Cl cotransporter in thick ascending limb (TAL) because of mutation in the *SLC12A1* gene on the 15q15-21 chromosome.[4,5]
2. BS, antenatal, type II, involving defect in ROMK (Renal Outer Medullary Potassium Channel) in TAL because of mutation in the *KCNJ1* gene on the chromosome 11q24.[6]
3. BS, classic, type III, involving defect in CIC-Kb channel in TAL and distal tubule because of mutation in the *CLCNKB* gene on the chromosome 1p36.[5]
4. BS type IV, BSND variant occurs because of mutation in the *BSND* gene on chromosome 1p31 coding for protein “Barttin” which forms the β subunit of CICKb and CICKa channels located on the basolateral membrane of TAL and inner ear epithelium.[7]
5. Gitelman's or hypomagnesemic variant[8] involving defect in NCCT channels in the distal tubule because of mutation in the *SLC12A3* gene on chromosome 16q13.[5]

Signs and symptoms of antenatal BS may be identifiable *in utero*. Antenatally, there is unexplained polyhydramnios and premature delivery. Amniotic fluid biochemistry shows normal sodium, potassium, prostaglandin levels but persistently increased chloride and aldosterone levels.[9]

Phenotypic features such as triangular facies, characterized by a prominent forehead, large eyes, strabismus, protruding ears, and a drooping mouth have been reported.[10] After birth, infants develop polyuria, lethargy, poor feeding, and rapid weight loss. Urine output increases up to 10 mL/ kg/h with hyposthenuria. There is increased urinary loss of sodium, potassium, chloride, and prostaglandin. Hematological investigation shows hyponatremia, hypokalemia, and hypochloremia with metabolic alkalosis; hypercalciuria with nephrocalcinosis also occurs. Blood levels of renin, aldosterone, and prostaglandin E_2_ are high and important in establishing the diagnosis.

The defects in the tubular channel cause increased loss of salts in urine, which leads to the activation of the renin-angiotensin-aldosterone axis, in turn, leading to hyperaldosteronism and hyperreninemia. The exact mechanism of increased prostaglandin levels in blood and urine is not known, but it appears to be secondary to the underlying defect in the transport of sodium chloride in TAL.[11] Thus, the term, “Hyperprostaglandin E syndrome” for these tubulopathies is a misnomer. However, why these patients with high renin and aldosterone levels have normal blood pressure is still not clear.

Type IV BS or BSND variant occurs due to mutation in the *BSND* gene coding for Barttin protein forming the β subunit of the basolateral chloride channel, CICKb, on TAL. These channels also contribute to endolymph secretion in the inner ear. Karl *et al*. reported a BSND variant case with digenic mutations in the CICKa and CICKb channels.[12] Such BSND variants have all other features of antenatal BS and these infants have high chances of neonatal infection compared to their degree of prematurity.

Classic BS presents at a later age with failure to thrive, polyuria, polydipsia, salt craving, and dehydration. History of polyhydramnios and prematurity is absent. Urinary calcium is normal and nephrocalcinosis is usually absent.

Gitelman's variant also has a milder course and later age of onset. Patients present with fatigue, muscle weakness, and recurrent episodes of tetany in the form of carpopedal spasm. History of polyhydramnios and prematurity is absent. They have hypomagnesemia and hypocalciuria.

Antenatal diagnosis is by mutational analysis of genomic DNA from cultured amniocytes obtained by amniocentesis at the 18^th^ week of gestation.[13]

Based on the theory of hyperprostaglandism, antenatal and postnatal treatment with indomethacin has shown promising results. A low dose of indomethacin, 0.5 mg/kg/dose, every 12 hours from 26 to 31 weeks of gestation is sufficient to arrest the progression of polyhydramnios. Indomethacin therapy should be monitored by fetal echocardiography and maternal serum indomethacin levels for complications such as premature closure of ductus arteriosus, oliguric renal dysfunction, and necrotizing enterocolitis.[13]

Fluid and electrolytes should be postnatally replaced according to the extent of the loss. Indomethacin should be started in a low dose (0.2 mg/kg/day). Close monitoring of serum creatinine, urinary prostaglandin, and serum indomethacin levels is mandatory to detect drug toxicity and response to therapy. The dose of indomethacin can then be titrated to achieve an adequate response. The indomethacin therapy has shown to decrease polyuria, renal salt-wasting, hyperprostaglandinuria, hypercalciuria, and nephrocalcinosis.
